# Exploring the impact of nonverbal social behavior on learning outcomes in instructional video design

**DOI:** 10.1038/s41598-024-63487-w

**Published:** 2024-06-04

**Authors:** Jonas Frenkel, Anke Cajar, Ralf Engbert, Rebecca Lazarides

**Affiliations:** 1 Department of Educational Sciences, University of Potsdam, Karl-Liebknecht-Straße 24/25, 14476 Potsdam, Germany; 2 Science of Intelligence, Research Cluster of Excellence, Marchstraße 23, 10587 Berlin, Germany; 3 Department of Psychology, University of Potsdam, Karl-Liebknecht-Straße 24/25, 14476 Potsdam, Germany

**Keywords:** Human behaviour, Psychology

## Abstract

Online education has become increasingly popular in recent years, and video lectures have emerged as a common instructional format. While the importance of instructors’ nonverbal social cues such as gaze, facial expression, and gestures for learning progress in face-to-face teaching is well-established, their impact on instructional videos is not fully understood. Most studies on nonverbal social cues in instructional videos focus on isolated cues rather than considering multimodal nonverbal behavior patterns and their effects on the learning progress. This study examines the role of instructors’ nonverbal immediacy (a construct capturing multimodal nonverbal behaviors that reduce psychological distance) in video lectures with respect to learners’ cognitive, affective, and motivational outcomes. We carried out an eye-tracking experiment with 87 participants (M_age_ = 24.11, SD = 4.80). Results of multilevel path analyses indicate that high nonverbal immediacy substantially increases learners’ state motivation and enjoyment, but does not affect cognitive learning. Analyses of learners’ eye movements show that learners allocate more attention to the instructor than to the learning material with increasing levels of nonverbal immediacy displayed by the instructor. The study highlights the importance of considering the role of multimodal nonverbal behavior patterns in online education and provides insights for effective video lecture design.

## Introduction

Online education has steadily increased in popularity over the past decade as technology has advanced and the need for flexible learning environments has grown^1^. In particular, online video lectures have become increasingly common in recent years^2^. While video lectures often differ in their design aspects, one of the main design considerations is the role of the instructor in the video^3^. In face-to-face interactions, the way in which instructors communicate nonverbally with their students is crucially important for effective knowledge transfer^4^. Consequently, researchers have recently begun to investigate the impact of an instructor’s nonverbal social cues such as gaze, facial expression, and gestures on the effectiveness of instructional videos^5^. The majority of studies report that video lectures with an instructor present reduce distraction and contribute to performance gains^6,7^. These studies often focus on analyzing the effects of specific individual behaviors, such as pointing^8^ or gaze^5^. Social cues, however, are a unified percept, and thus need to be considered as an integrated whole^9–11^. The present study addresses this research gap by examining the role of instructors’ nonverbal immediacy in video lectures with respect to learners’ cognitive, affective, and motivational learning outcomes. Nonverbal immediacy refers to a comprehensive and multidimensional construct that captures nonverbal behaviors aimed at reducing psychological distance, such as eye contact, relaxed and open body posture, gesturing, and smiling.

Past research on the design of multimedia learning formats has primarily adopted a cognitive perspective and has mostly been guided by the idea that the amount and type of information students process during instruction can play an important role in their learning^12^. In this context, cognitive load theory of multimedia learning (CLTM)^13^ is referenced as a theoretical approach that offers a conceptual model for understanding the cognitive processes involved in instruction. CLTM identifies three types of cognitive load that compete for working memory resources: (i) Intrinsic load refers to the complexity of the material being learned, (ii) extraneous load refers to the mental effort required by learning activities and their design, and (iii) germane load refers to the mental effort learners expend to integrate new information into their existing knowledge structures^14^. In this framework, effective learning materials should aim to minimize extraneous load, enabling learners to allocate most mental resources to germane load. Consequently, additional visual features in online learning settings may distract learners from processing the instructional content, resulting in a split-attention effect^15^. This seems even more likely in the case of nonverbal cues that do not have semantic meaning^16^. More recently, however, this model has been criticized as it does not explain why some forms of seemingly irrelevant nonverbal cues, such as gesturing and pointing, foster learning outcomes^17^.

Research on effective learning and teaching in online learning settings also emphasizes the role of affective-motivational processes and social factors^18^. Social agency theory^19^ suggests that the use of nonverbal cues in video lectures can reinforce learners’ sense that they are interacting with a “real” person, creating the impression of increased social presence^20^. The feeling of social presence can strengthen a social connection between the learner and the instructor^21^, eliciting positive socio-emotional responses^22,23^. Increased social presence thus leads the students to be more attentive^24^, motivating them and engaging them in deeper cognitive processing of the material^25^. Moreover, an increased social presence of the instructor can result in enhanced positive affective responses^26,27^. Thus, social cues can elicit positive social responses and promote learners’ motivation and understanding. However, it is currently unclear whether the instructor’s presence as an additional visual stimulus may not also distract learners and reduce attention and learning^28^.

In order to examine the factors that influence the complex interplay between cognitive and affective-motivational processes in social (online) learning settings, certain gaps in existing research need to be addressed: Firstly, in the context of teachers’ nonverbal behavior, previous research has primarily followed a reductive approach that focuses on a limited selection of specific nonverbal cues, while tending to analyze these cues in isolation and neglect their interrelationships^29^. Nonverbal communication, however, relies on the complex interplay of multiple nonverbal cues expressed in conjunction with one another. In this context, the effects of individual cues are not simply additive, and certain nonverbal cues can change their meaning and intensity when combined^29^. Especially with respect to affective-motivational processes, all movements made by an instructor could be effective as social cues and thus influence learning performance^21^. To account for this reduced decomposability, it is crucial to study the combined effects of nonverbal cues in addition to examining the effects of individual cues. Secondly, most studies adopt a rather simplistic research design in which these specific nonverbal cues are either present or absent. However, since the effects of teachers’ nonverbal behavior may not always be strictly linear^9,30^, going beyond the presence or absence of nonverbal cues to examine responses to their varying intensities would be of additional interest.

A construct that is suitable for describing nonverbal behavior in a multidimensional and complex way by capturing behaviors in varying degrees of intensities is nonverbal immediacy (NVI). Introduced by Mehrabian^31^, NVI encompasses behaviors that convey positive signals to the communication partner, such as sympathy and warmth, thereby reducing the psychological distance between instructors and students. Typical NVI behaviors include gestures, eye contact, relaxed posture, or smiling^32,33^. Meta-analyses have found positive associations between levels of NVI and various learning outcomes in real-life settings, including recall of learned material, attitudes toward content, and course or student motivation^32,34^. In the online teaching context, however, NVI behaviors remain under-researched^35^. The inclusion of this construct could be helpful in filling some of the gaps in previous research on the effects of nonverbal cues and in providing more comprehensive and detailed recommendations for video lecture design.

**Figure 1:**
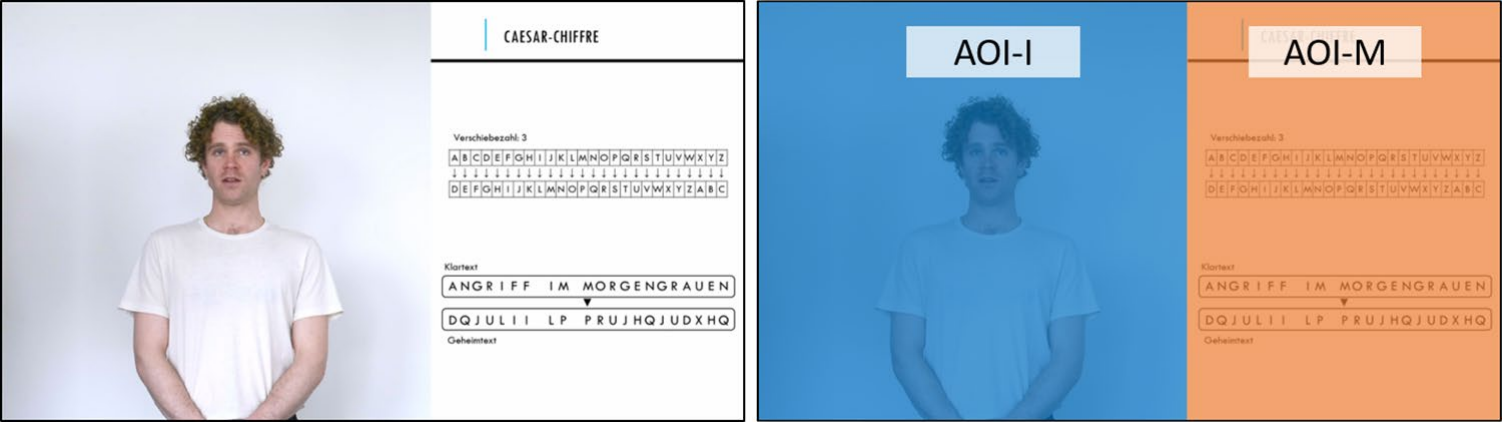
(**a**) Screenshot of the experimental stimulus videos. All videos showed an actor playing the role of an instructor and the corresponding instructional material. (**b**) Illustration of the defined areas of interest *instructor* (AOI-I) and *material* (AOI-M).

The aims of the current study were (i) to investigate the relations between three different intensities of instructors’ NVI in video lectures and students’ cognitive, affective, and motivational outcome measures, and (ii) to analyze the extent to which instructors’ NVI is related to the allocation of students’ attention between multiple relevant sources of information. To this end, participants watched four educational videos on cryptology while their eye movements were tracked. Videos displayed learning material on one half of the screen and an instructor on the other half of the screen (see Fig. 1a), with the instructor expressing one of three possible levels of NVI (low, medium, high). The amount of participants’ perceived NVI as well as its effects on their cognitive learning success and affective and motivational factors were ascertained through pre- and post-test questionnaires (for details, see “Methods” section).

We tested the following hypotheses:

- We expected perceived NVI to be lowest in the condition in which the instructor displayed the lowest level of immediacy-related nonverbal behaviors in the videos (low condition) and highest in the condition in which the instructor displayed the highest level of immediacy-related nonverbal behaviors (high condition; H1a). Furthermore, we hypothesized that cognitive learning success (H1b), state motivation (H1c), and state enjoyment (H1d) would correlate positively with the level of perceived NVI.
- Regarding the effects of NVI on eye-movement behavior, we hypothesized that﻿ the time spent looking at the instructor versus the learning material in the video would differ depending on the instructor’s level of immediacy. Specifically, we expected the relative number of fixations on (H2a) and relative total viewing time (H2b) directed at the instructor to increase with increasing levels of instructor NVI.

## Results

### Descriptive statistics

Descriptive statistics are reported in Table 1. Bivariate correlations are shown in Table 2. Perceived nonverbal immediacy was positively and substantially correlated with state motivation and state enjoyment. For the trait variables, correlations were substantial and positive between trait motivation, trait enjoyment, and both of their state measures. No substantial correlations were found between cognitive learning and any of the other outcome variables.

**Table 1:** Descriptive statistics of the study variables.

	Total	SD	α	Low condition		Medium condition		High condition	
	M			M	SD	M	SD	M	SD
State motivation Video 1	5.02	1.08	0.78	5.26	1.24	4.98	0.90	4.92	1.10
State motivation Video 2	5.05	1.21	0.85	5.52	1.01	4.95	0.98	4.77	1.44
State motivation Video 3	5.06	1.31	0.84	5.43	1.36	4.88	1.08	5.00	1.41
State motivation Video 4	4.99	1.40	0.89	5.31	1.40	4.91	1.20	4.83	1.56
(Range 1–7)									
State enjoyment Video 1	4.65	1.18	0.90	4.85	1.26	4.59	1.03	4.53	1.27
State enjoyment Video 2	4.66	1.23	0.92	5.17	1.13	4.54	0.95	4.35	1.42
State enjoyment Video 3	4.64	1.25	0.89	5.05	1.31	4.53	1.02	4.43	1.38
State enjoyment Video 4	4.61	1.35	0.91	5.08	1.34	4.39	1.15	4.41	1.49
(Range 1–7)									
Trait motivation	5.05	0.95	0.79	5.37	0.74	4.95	0.89	4.85	1.14
Trait enjoyment	4.93	1.05	0.89	5.26	0.95	4.75	1.12	4.87	1.04
(Range 1–7)									
Cognitive learning	5.62	1.73		5.68	1.56	5.89	1.95	5.32	1.68
(Range 0–18)									
Perceived NVI	66.92	11.84	0.90	63.48	12.70	67.89	11.25	70.10	10.55
(Range 18–90)									
Total viewing time AOI-I (part 1)	15.99	5.36		15.46	5.49	16.07	4.33	16.39	6.14
Total viewing time AOI-I (part 3)	56.54	15.61		52.08	13.75	56.14	13.06	60.93	18.35
(in s)									
Relative viewing time AOI-I (part 1)	0.65	0.10		0.63	0.11	0.64	0.10	0.67	0.09
Relative viewing time AOI-I (part 3)	0.59	0.08		0.54	0.08	0.58	0.07	0.64	0.08
Total number of fixations AOI-I (part 1)	36.07	14.06		35.71	14.86	34.02	12.66	38.26	14.65
Total number of fixations AOI-I (part 3)	152.56	43.86		142.92	45.41	149.14	41.57	164.35	43.16
Relative number of fixations AOI-I (part 1)	0.52	0.10		0.51	0.10	0.50	0.11	0.56	0.10
Relative number of fixations AOI-I (part 3)	0.52	0.08		0.48	0.06	0.52	0.07	0.57	0.08

*NVI* nonverbal immediacy, *AOI-I* area of interest-instructor.

**Table 2:** Bivariate correlations of the study variables.

		(1)	(2)	(3)	(4)	(5)	(6)	(7)	(8)	(9)	(10)	(11)	(12)
(1)	State motivation Video 1	–											
(2)	State motivation Video 2	0.69**	–										
(3)	State motivation Video 3	0.56**	0.85**	–									
(4)	State motivation Video 4	0.57**	0.82**	0.86**	–								
(5)	State enjoyment Video 1	0.72**	0.79**	0.71**	0.75**	–							
(6)	State enjoyment Video 2	0.65**	0.91**	0.80**	0.81**	0.82**	–						
(7)	State enjoyment Video 3	0.56**	0.82**	0.91**	0.88**	0.74**	0.86**	–					
(8)	State enjoyment Video 4	0.57**	0.82**	0.85**	0.92**	0.75**	0.87**	0.90**	–				
(9)	Trait motivation	0.34**	0.43**	0.40**	0.38**	0.46**	0.43**	0.43**	0.46**	–			
(10)	Trait enjoyment	0.22*	0.33**	0.38**	0.32**	0.46**	0.38**	0.38**	0.37**	0.74**	–		
(11)	Cognitive learning	0.05	0.08	0.03	0.06	0.05	0.02	− 0.04	0.04	0.04	− 0.14	–	
(12)	Perceived nonverbal immediacy	0.27*	0.28**	0.36**	0.39**	0.46**	0.35**	0.35**	0.36**	0.14	0.14	− 0.02	–

*p < 0.05; **p < 0.01.

### Perceived nonverbal immediacy and cognitive learning

To investigate the association between perceived NVI and experimental condition (H1a), a baseline path model (Model 0) was established. The baseline model was saturated, χ^2^ = 0.00, df = 0, CFI = 1.00, TLI = 1.00, RMSEA = 0.00, SRMR = 0.00. Results indicated a substantial effect of experimental condition on perceived NVI for the high compared to the low condition, but not for the medium compared to the low condition (H1a). In Model 1, cognitive learning was added as an outcome variable, while gender, language skills, general cognitive performance, effort, and prior knowledge on the topic of cryptology were included as predictors. Results of the path analyses in Model 1 are reported in Fig. S3 and Table 3. The model showed a good fit to the data, χ^2^ = 6.36, df = 8, CFI = 1.00, TLI = 1.00, RMSEA = 0.00, SRMR = 0.06. Again, experimental condition had a substantial effect on perceived NVI for the high compared to the low condition, but not for the medium condition. Participants in the high condition reported substantially higher perceived NVI than did participants in the low condition. No substantial relation between perceived NVI and cognitive learning could be found (H1b), but age and perceived effort were significantly related to cognitive learning (*R*^2^ = 0.19, *p* = 0.04).

**Table 3:** Standardized regression coefficients for cognitive learning.

	Cognitive learning				Perceived NVI			
	β	SE	95% CI	p	β	SE	95% CI	p
Experimental condition								
Medium condition					0.19	0.13	[− 0.06, 0.45]	0.13
**High condition**					**0.30**	**0.12**	**[0.07, 0.54]**	**0.01**
Perceived NVI	− 0.05	0.11	[− 0.26, 0.17]	0.67				
Age	− **0.22**	**0.12**	**[**− **0.45,** − **0.03]**	**0.04**				
Male	0.00	0.12	[− 0.20, 0.20]	0.98				
WAIS-DSST	**0.17**	**0.10**	**[**− **0.04, 0.37]**	**0.12**				
MWT	0.01	0.09	[− 0.16, 0.19]	0.87				
Effort	− **0.29**	**0.12**	**[**− **0.51,** − **0.01]**	**0.01**				
Prior knowledge	0.00	0.12	[− 0.23, 0.17]	0.98				
(*R*^2^ = 0.35, *p* < 0.01)								

*NVI* nonverbal immediacy, *WAIS-DSST* Wechsler adult intelligence scale-digit-symbol subtest, *MWT* Mehrfachwahl–Wortschatz–Intelligenz test (multiple-choice vocabulary intelligence test).

Significant values are in bold.

### Perceived nonverbal immediacy: state motivation and state emotion

Building on the baseline model, Model 2 was specified to assess the relationship between perceived NVI and state motivation. On the trial level (L1), the model included measures of the outcome variables after each of four trials. On the subject level (L2), latent aggregates of state motivation were added as an outcome variable and perceived NVI and experimental condition were included as predictors, while controlling for trait motivation, age, gender, language skills, and general cognitive performance. The results of the multilevel path analysis in Model 2 (H1c) are reported in Fig. S4 and Table 4. The model showed a good fit to the empirical data, χ^2^ = 52.99, df = 50, CFI = 0.99, TLI = 0.99, RMSEA = 0.01, SRMR_within_ = 0.03, SRMR_between_ = 0.09. The multilevel analyses showed that, on the trial level (L1), no substantial association between the state motivation and the trial number could be found. On the subject level (L2), participants in the high condition reported substantially higher perceived NVI scores than did participants in the low condition, but not compared to the medium condition. State motivation was positively related to perceived NVI when controlling for trait motivation. On the subject level, but not on the trial level, the model explained a significant amount of variance in state motivation (*R*^2^ = 0.39, *p* < 0.01).

**Table 4:** Standardized regression coefficients for state motivation.

	State motivation				Perceived NVI			
	β	SE	95% CI	p	β	SE	95% CI	p
Subject level								
Experimental condition								
Medium condition					0.13	0.13	[− 0.12, 0.38]	0.31
**High condition**					**0.30**	**0.12**	**[0.07, 0.52]**	**0.01**
Perceived NVI	**0.23**	**0.11**	**[0.01, 0.44]**	**0.04**				
Trait motivation	**0.53**	**0.12**	**[0.30, 0.76]**	**0.00**				
Age	− 0.17	0.11	[− 0.38, 0.04]	0.11				
Male	0.04	0.12	[− 0.19, 0.28]	0.71				
WAIS-DSST	− 0.05	0.10	[− 0.24, 0.15]	0.64				
MWT	− 0.15	0.12	[− 0.38, 0.08]	0.19				
(*R*^2^ = 0.39, *p* < 0.01)	0.39							
Trial level								
Trial	0.02	0.08	[− 0.15, 0.18]	0.86				
(*R*^2^ = 0.00, *p* = 0.93)								

*NVI* nonverbal immediacy, *WAIS-DSST* Wechsler adult intelligence scale-digit-symbol subtest, *MWT* Mehrfachwahl–Wortschatz–Intelligenz test (multiple-choice vocabulary intelligence test).

Significant values are in bold.

Model 3 was specified building on the baseline model to examine the relationship between perceived NVI and state enjoyment. At the trial level (L1), measures of outcome variables after each of the four trials were incorporated into the model. Latent aggregates of state enjoyment were included as outcome variables at the subject level (L2). Perceived NVI and experimental condition were included as predictors, while controlling for trait enjoyment, age, gender, language proficiency, and general cognitive performance. The results of Model 3 (H1d) are reported in Fig. S5 and Table 5. The model showed an acceptable fit to the empirical data, χ^2^ = 109.03, df = 82, CFI = 0.96, TLI = 0.95, RMSEA = 0.03, SRMR_within_ = 0.05, SRMR_between_ = 0.09. On the trial level (L1), the reported state enjoyment was not substantially related to the trial number. On the subject level (L2), participants in the high condition reported substantially higher perceived NVI scores than participants in the low condition, but not compared to the medium condition. Perceived NVI showed a positive relation to state enjoyment when controlling for trait enjoyment. The model explained a significant amount of variance in participants’ state enjoyment on the subject level (*R*^2^ = 0.28, *p* < 0.01).

**Table 5:** Standardized regression coefficients for state enjoyment.

	State enjoyment				Perceived NVI			
	β	SE	95% CI	p	β	SE	95% CI	p
Subject level								
Experimental condition								
Medium condition					0.13	0.13	[− 0.12, 0.38]	0.31
High condition					**0.30**	**0.12**	**[0.07, 0.52]**	**0.01**
Perceived NVI	**0.23**	**0.11**	**[0.01, 0.52]**	**0.04**				
Trait enjoyment	**0.40**	**0.12**	**[0.17, 0.64]**	**0.00**				
Age	− **0.26**	**0.10**	**[**− **0.46,** − **0.05]**	**0.01**				
Male	− 0.02	0.11	[− 0.24, 0.21]	0.89				
WAIS-DSST	0.01	0.11	[− 0.21, 0.22]	0.95				
MWT	− 0.03	0.10	[− 0.23, 0.16]	0.73				
(*R*^2^ = 0.28, *p* < 0.01)	0.28							
Trial level								
Trial	− 0.04	0.08	[− 0.20, 0.12]	0.61				
(*R*^2^ = 0.00, *p* = 0.80)								

*NVI* nonverbal immediacy, *WAIS-DSST* Wechsler adult intelligence scale-digit-symbol subtest, *MWT* Mehrfachwahl–Wortschatz–Intelligenz test (multiple-choice vocabulary intelligence test).

Significant values are in bold.

### Eye movements

**Figure 2:**
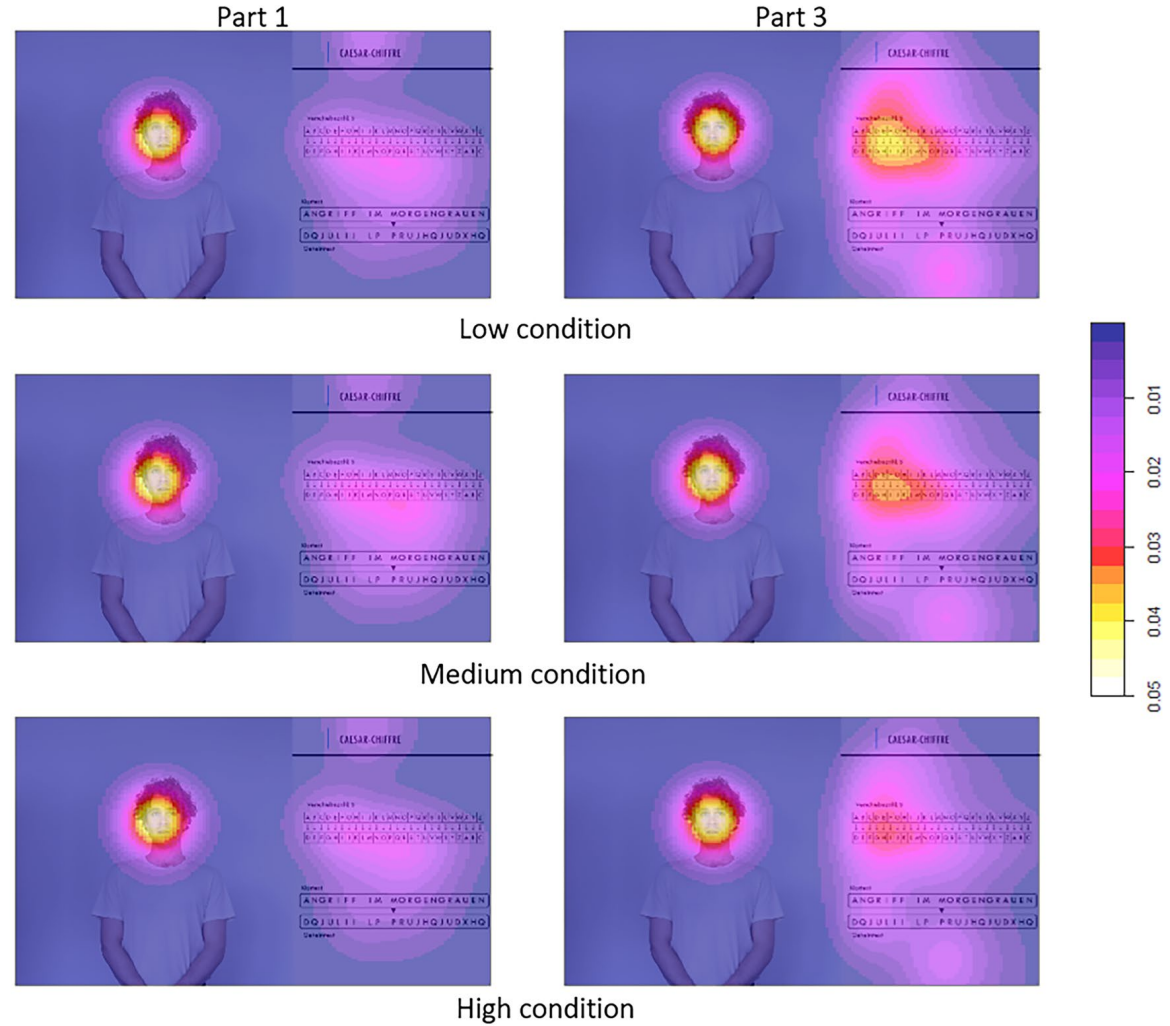
Kernel density plot of fixations on the stimulus material during parts 1 and 3 for all videos in each experimental condition.

Each of the videos was divided into three parts: a 40-s baseline phase (part 1) that was identical for all conditions and in which the instructor displayed a medium level of NVI-related behaviors, a 20-s transition phase to mask sudden changes in the level of NVI displayed (part 2), and an approximately 120-s phase in which the level of NVI-related behaviors displayed by the instructor was manipulated according to the experimental condition (part 3) (for more information, see the “Methods” section). Descriptive statistics on the relative number of fixations on and relative viewing time directed at the instructor (H2a + H2b) for parts 1 and 3 across all videos are included in Table 1 (for a full description of all parts and videos, see Table S4a,b). Figure 2 shows the distribution of fixations between experimental conditions during parts 1 and 3 of the videos. As expected, differences in fixation locations were observed mainly during part 3 of the videos, whereas the density of fixations was found to be comparable across all conditions during part 1. Between-group comparison of the relative number of fixations on target area AOI-I during parts 1 and 3 of the videos by multivariate ANOVA revealed a significant effect of experimental condition (*F*(2, 83) = 6.25, *p* < 0.01) as well as a significant interaction effect between condition and video parts (*F*(2, 83) = 4.07, *p* = 0.02). Pairwise post-hoc comparisons (alpha levels adjusted by Bonferroni Holm) showed that the mean relative number of fixations on the instructor during part 3 of the videos was substantially higher in the high condition, compared to both the medium (*p*_*adj*_ = 0.02) and low (*p*_*adj*_ < 0.01) conditions. The relative number of fixations on target area AOI-I during part 1 of the videos did not differ substantially between the conditions. An analysis of the relation between the relative number of fixations on target area AOI-I and perceived NVI revealed a significant positive correlation during part 3 (*r* = 0.19, *p* < 0.01) but not during part 1 (*r* = 0.06, *p* = 0.27).

A multivariate ANOVA of the relative duration of fixations on the instructor during the first and third parts of the videos revealed a significant main effect of both experimental condition (*F*(2, 83) = 5.28, *p* < .01) and video parts (*F*(1, 83) = 55.92, *p* < 0.01), as well as a significant interaction between condition and video parts (*F*(2, 83) = 3.47, *p* = 0.03). Post-hoc comparisons (alpha levels adjusted by Bonferroni Holm) revealed significantly longer fixation durations for the high condition compared to the low condition (*p*_*adj*_ < 0.01) and the medium condition (*p*_*adj*_ = 0.03) during part 3 of the videos. No substantial group differences were found for the first part of the videos. No significant correlations were found between cognitive learning and relative durations of fixation on the instructor (*r* = −.25, *p* = 0.65).

## Discussion

Although instructional videos for online education are widely employed, there is still no conclusive understanding of how instructors in those videos should behave. The present study investigated how an instructor’s nonverbal immediacy, which reduces psychological distance between learner and instructor, affects cognitive and affective-motivational learning outcomes as well as the allocation of learners’ attention. The main findings of our study are that instructors’ nonverbal behaviors matter for learners’ state motivation and enjoyment in instructional videos. Compared to low NVI, high levels of NVI were related to higher state motivation and emotion, but not to better cognitive learning outcomes during learning. Further, instructors’ nonverbal immediacy also affected attention, as the relative number of fixations on and the relative viewing time directed at the instructor were particularly high when NVI was high. However, differences in terms of cognitive learning success could not be found. In the following sections, we discuss these findings along with our hypotheses.

In line with our assumptions (H1a), our findings show that the intensity and number of nonverbal social cues displayed by the instructor in the different video conditions are relevant for learners’ perceived NVI. Thus, similar to face-to-face interaction, some types of nonverbal behavior in a video setting appear to be effective in reducing the psychological distance between instructor and learner, and the construct of NVI seems to be capable of reflecting these changes.

Contrary to the expectations derived from social action theory (H1b), our findings do not provide any evidence of an increase in cognitive learning associated with nonverbal immediacy. This aligns with previous studies conducted in the field of video instructional research, which have also failed to establish a significant relationship between instructors’ nonverbal behavior and cognitive learning^5^. Additionally, research conducted in real-world settings has shown a much weaker relationship between NVI and cognitive learning compared to affective learning outcomes^34^. One plausible explanation for the lack of significant effects of nonverbal social behavior on cognitive learning could be that nonverbal immediacy indirectly influences cognitive learning through affective-motivational processes. This notion finds support in several studies that, employing path analyses, indicate that instructor immediacy might enhance students’ state motivation^36–38^ and evoke positive emotional responses in students^39,40^, which then in turn contributes to increased cognitive learning.

Furthermore, it is worth noting, that the design of the teaching materials in this study was based on a university context. In higher education, the nature of learning is multifaceted, and instructional videos represent just one element of a broader pedagogical framework, that encompasses an extensive set of educational activities aimed at fostering cognitive activation and self-directed learning. This complexity may account for the lack of significant effects of NVI on cognitive learning outcomes in our study. In other contexts, such as schools, where students rely more on structured instruction, NVI may have a more pronounced impact on cognitive learning outcomes.

Nevertheless, some studies have reported positive effects of nonverbal instructor cues on cognitive learning outcomes. For example, Pi et al.^41^ and Stull et al.^42^ found increased post-test scores in students who watched instructional videos with instructors displaying nonverbal cues. The inconclusive results in this regard could also be attributed to the specific instructional content used. Pi et al.^21^, for example, highlight that the effects of nonverbal instructor cues on cognitive learning were particularly pronounced for easier content. Another explanation is that most studies reporting positive associations of NVI with cognitive learning are limited to a few types of nonverbal cues such as pointing and gaze^42,43^. It is possible that limited experimental designs allow attention-directing effects to have a stronger impact, resulting in stronger effects on cognitive learning performance.

In line with our expectations (H1c + d), the results indicate a positive relation between the instructor’s nonverbal social behavior and learners’ affective-motivational learning outcomes. Across all videos, learners’ state motivation and state enjoyment were positively associated with perceived NVI. Our findings thus align with the assumptions of social agency theory^19^, suggesting that nonverbal cues can elicit positive socio-emotional responses, thereby enhancing the connection between instructors and students and promoting engagement. Based on our findings, it is reasonable to assume that the learner-perceived NVI of an instructor may be a key factor in promoting motivational-affective development in learning environments that utilize instructional videos.

Furthermore, while our findings are situated within the context of asynchronous instructional videos, they also indicate the potential significance of NVI in synchronous online learning environments. In synchronous settings, where real-time interaction enhances engagement, the benefits of high NVI may be even more pronounced. The reduced psychological distance and more favorable perceptions of the instructor facilitated by high NVI could contribute to increased engagement during sessions and a greater willingness among students to ask questions and participate actively. These advantages highlight the broader relevance of NVI, suggesting that it could potentially be leveraged effectively across various online learning settings.

Our eye-tracking measures revealed a correlation between the NVI of the instructor and learners’ attention allocation. In relevant video segments, the high-NVI group demonstrated, as expected (H2a + b), a substantially higher number of fixations on and longer viewing times directed at the instructor compared to the low-NVI group. These findings confirm prior research by Kizilcec et al.^7^ and Wang et al.^43^, who also observed a strong focus of attention on the instructor. Results suggest that increased nonverbal behavior by the instructor may divert attention from relevant instructional content, potentially leading to an attentional split and increased cognitive load. However, despite the differences in the distribution of attention, we did not find substantial negative effects on cognitive learning, as would be suggested by cognitive load theory^13^. Further, no substantial correlation was found between fixation time spent on the instructional material versus the instructor and cognitive learning. Thus, the assumption put forward by the CLTM that maximizing the time spent looking at the learning material increases cognitive learning, while diverting attention to the instructor results in learning loss, could not be confirmed. Moreover, examining the temporal course indicated systematic variations in attentional focus across all participants, independent of the intensity of nonverbal instructor behavior. This indicates a substantial relevance of the instructional content viewed, such that learners direct their attention to the necessary instructional material at relevant points, even when the instructor displays high NVI.

Several limitations of this study should be acknowledged, pointing to directions for future research. Firstly, it is important to note that our study was designed to analyze the instructor’s nonverbal social behavior as a holistic construct, rather than to dissect individual nonverbal cues. Consequently, our analysis employed relatively broad AOIs with limited temporal and spatial resolution. While an investigation into the interactions between specific nonverbal cues would undoubtedly be insightful, such an exploration was beyond the research questions of the present investigation and will be addressed through more granular analyses in future publications. Secondly, the levels of NVI were not constant within each experimental condition, but varied throughout each video. While this design ensured a baseline measurement for the eye-tracking data, it may have attenuated potential group differences with respect to various learning outcomes. Thirdly, the relatively invasive method of eye-tracking using a head-chin rest might also impact the ecological validity of the results. For example, participants were able to focus their attention on only the instructor or the material, but not on external stimuli, which would be expected to be more prevalent in an online learning environment.

In conclusion, the present study indicates that an instructor’s nonverbal immediacy benefits affective-motivational learning outcomes for learners watching instructional videos. Furthermore, we demonstrate that an increased intensity of NVI leads to a stronger focus of learners’ visual attention on the instructor. However, the level of NVI does not substantially affect cognitive learning outcomes. Thus, including nonverbal social cues in instructional video designs might foster learners’ motivation and positive emotions without necessarily reducing their cognitive learning outcomes.

## Methods

### Sample description

This study was performed in line with the principles of the Declaration of Helsinki and received approval from the University of Potsdam Ethics Committee (number 27/2023). Informed consent was obtained from all participants prior to participation. Individuals who are identifiable in the images and video gave informed consent for publication in an open-access online publication. Experimental data were collected from a total of N = 102 university students. Due to technical complications that resulted in partial questionnaire data loss, 15 participants had to be excluded from the study, resulting in N = 87 (female= 61, male = 23, non-binary = 3) valid data sets. The age of the participants ranged from 18 to 38 years (M = 24.11, SD = 4.80). Participants had normal or corrected-to-normal vision. Participants were German-speaking, with 13 participants reporting that they were not native speakers. The majority of participants were undergraduate students (N = 67), while N = 11 participants reported that they were pursuing a master’s degree, and N = 9 subjects were doctoral students. Most of the subjects either pursued a teaching degree (N = 32) or majored in psychology or cognitive science (N = 32). Participants who were not required to participate in experiments as part of their educational program were compensated with an amount of EUR 20.

### Procedure

Prior to the commencement of the study, a pilot study was conducted to validate the experimental design, with a particular focus on the instructional videos and the cognitive learning items. For this purpose, the experiment was completed by a total of 5 students. Feedback was gathered through questionnaire comments and verbal debriefings. Results indicated that the design of the study was adequate, the questions were appropriate in number, content, and difficulty, the non-verbal behavior of the instructor was perceived as natural across all experimental conditions and the videos effectively met the educational objectives. Consequently, no significant modifications to the study design were required.

Participants were randomly assigned to three experimental conditions corresponding to different intensities of NVI behavior demonstrated by the instructor in the video (low condition = 27, medium condition = 29, high condition = 31). Participants were naïve about the specific purpose of the experiment and were instead informed in general terms that the study was conducted to investigate the influence of various factors on the effectiveness of instructional videos. A visual overview of the experimental procedure can be found in Fig. S1. The total duration of the experiment was 80 min. In the pre-experimental phase (40 min), the participants’ visual acuity was verified using the Freiburg Visual Acuity Test^44^. In addition, a color discrimination test was performed and the stereoscopic vision of the participants was verified. Sociodemographic data were then collected and participants’ trait motivation and trait enjoyment were assessed. Prior knowledge of the instructional content was evaluated using a single-item self-report.

The main experimental phase (40 min) consisted of four trials (trial 1–4). During each trial, participants watched one instructional video on the topic of cryptology (approx. 3 min). After the video, participants’ cognitive learning was measured using multiple choice questions. An overview of the topics presented in the videos, as well as example questions are reported in Table S1. Participants then filled in a short survey, assessing their state motivation, and their state enjoyment, before progressing to the next trial.

After completing all four trials, the instructor’s level of NVI was rated by the participants. Moreover, participants were asked to indicate the perceived effort involved in watching the instructional videos. Videos were designed to resemble a typical teaching format in lectures at German universities, which commonly consist of a lecturer supplemented by PowerPoint slides. To this end, the screen was divided into two sections, with an instructor featured on the left side of the screen and additional learning material presented on the right side (see Fig. 1a).

Each video consisted of a total of three phases (parts 1–3) varying in the intensity of NVI behaviors displayed by the instructor. To facilitate eye-tracking measurements, each video began with a 40-s baseline phase (part 1) that was identical across all experimental conditions and in which the NVI level was kept at an intermediate level. In the low and high conditions, a transition period of about 20 seconds followed in order to avoid abrupt changes (part 2, with a medium-low or medium-high NVI level, respectively), before the instructor’s nonverbal behavior transitioned to a very low or very high NVI level for the remainder of the video (part 3, about 2 minutes). In the medium condition, the instructor’s NVI-related nonverbal behavior remained at an intermediate level throughout all three parts of each video (see Fig. S2).

In order to manipulate the level of nonverbal immediacy displayed, the instructor, a professional actor, was instructed to progressively increase the intensity and frequency of nonverbal social cues across conditions. Given that the overall NVI level was to be adjusted holistically, the relevant cues were varied simultaneously rather than individually. The selection of these cues was guided by NVI literature to maximize the differences along this scale. Specifically, the instructor adjusted his posture (from upright and stiff to overtly relaxed), facial expressions (from low variety, predominantly neutral to pleasant and smiling), gesture intensity (from small, infrequent gestures to constant and elaborate gestures), and tone of voice (from monotone with slight variations to dynamic and varied). Table S2 details the allocation of the corresponding nonverbal behaviors to the individual conditions. Specifically, the instructor adjusted his posture (from upright and stiff to overtly relaxed), facial expressions (from expressionless to frequent smiling and high variety in expressions), gesture intensity (from no gestures to constant and expressive gestures), and tone of voice (from flat and monotone to animated and varied).

The video stimuli were presented on a 24-inch VIEWPixx monitor (screen resolution 1920 × 1080 pixels, refresh rate 120 Hz). Participants were seated in a dimly lit room at a viewing distance of 66 cm (26 inches) from the monitor with their head stabilized by a head-chin rest. Stimuli and response collection were controlled with MATLAB^45^ using the Psychophysics Toolbox^46,47^, which includes the Eyelink Toolbox^48^. Eye movements of the dominant eye were recorded using an EyeLink 1000 Tower Mount system (version 4.56; SR Research, Osgoode/Ontario, Canada) with a sampling rate of 1000 Hz and a spatial resolution better than 0.01°.

### Measures

#### Cognitive learning

Cognitive learning was assessed using 4 to 6 multiple-choice questions following each of the four videos. To ensure the questions formed a unidimensional set, a Confirmatory Factor Analysis (CFA) was conducted, with stepwise removal of items that did not significantly load on a single factor. Consequently, a total of eight multiple-choice questions were considered for subsequent analyses.

#### Student enjoyment

Trait enjoyment and state enjoyment were measured using the corresponding subscales of the Achievement Emotions Questionaire—Short Version (AEQ-S), developed by Bieleke et al.^49^. Each was assessed using four items on a 7-point scale (1 = strongly disagree to 7 = strongly agree).

#### Student motivation

Trait motivation was measured using the situational motivation scale^50^. The questionnaire includes a total of 16 items related to the four subscales of intrinsic motivation, identified regulation, external regulation, and amotivation. Responses were given using a 7-point scale (1 = doesn’t correspond at all to 7 = corresponds exactly). Following Angot^51^, students’ state motivation was measured by 2 items of the situational motivation scale, focused on interest- and enjoyment-related aspects of the intrinsic motivation.

#### Perceived nonverbal immediacy (NVI)

The perceived NVI of the instructor was evaluated using the nonverbal immediacy scale^52^. This widely validated scale (for an overview of studies see^32^) includes a number of items assessing how often a person displays specific NVI behaviors via a five-point scale (1 = never to 5 = very often). The original set of 26 items was reduced to 18 items by the exclusion of eight items pertaining to proxemics and socially appropriate touch, which were deemed to be inapplicable to the context instructional videos.

#### Additional measures

Students’ German language skills were assessed using the multiple-choice vocabulary intelligence test (Mehrfachwahl–Wortschatz–Intelligenztest, MWT)^53^. The general cognitive ability of the participants was measured with the Digit-Symbol subtest of the Wechsler Adult Intelligence Scale, German Version^54^. Perceived effort was assessed with the corresponding subscale of the NASA task load index, German version^55^. Prior knowledge on the topic of cryptology was judged by the participants using a five-point single item scale (1 = no knowledge at all to 5 = very extensive knowledge). Participants’ visual acuity was verified using the Freiburg Visual Acuity Test^44^.

### Statistical analysis

For the questionnaire part of the study, we examined the relations between the level of NVI teaching behavior and students’ cognitive, affective, and motivational characteristics using a series of separate path models. One model was tested for each of the outcome variables. Simultaneous implementation of all outcome variables was avoided to prevent a reduction in the statistical power of the model and to minimize the risk of overfitting. In order to test whether perceived NVI was related to experimental condition (H1a), a baseline path model (Model 0) was specified. In the next model (Model 1), the outcome variable of cognitive learning was added to the baseline model to test whether it was associated with perceived NVI (H1b). Additional variables were age, gender, language skills, general cognitive performance, effort, and prior knowledge on the topic of cryptology. In the interest of simplicity, covariates were not correlated in all models. Additional model series, where correlations among the covariates were included, revealed that these correlations did not have any impact on the model fit or coefficient estimates across the models. Finally, an indirect effect of experimental condition on cognitive learning mediated by perceived NVI was included.

In the next step, also building on the basic model, two multilevel models (Models 2 & 3) were specified. To assess the relation between perceived immediacy and state motivation (H1c) and state enjoyment (H1d), measures of the outcome variables after each of four trials (Level 1: L1) were nested within subjects (Level 2: L2). We included latent aggregates of state motivation and enjoyment at L2 as outcome variables predicted by NVI and experimental condition on L2. Prior to the analyses, we ensured that a substantial amount of variance in motivation (ICC = 0.73) as well as in state enjoyment (ICC = 0.82) was explained on the subject level. This part of the model was controlled for trait motivation, trait enjoyment, age, gender, language skills, and general cognitive performance. On Level 1 (trials), we tested whether state enjoyment was related to time (trial number).

Prior to hypothesis testing with multilevel modeling, we tested metric invariance across both levels for students’ state motivation as well as for students’ state enjoyment. Invariance testing followed the steps described by Byrne^56^, and cut-off criteria for measuring non-invariance were used in line with Chen^57^. Results are displayed in Table S3 of the Supplement and the results confirmed metric invariance, thus all factor loadings were equal across time for state enjoyment and state motivation.

Analyses were conducted using Mplus 8.8^58^. Maximum likelihood estimation with robust standard errors was used. Missing data were handled using full information maximum likelihood. Model fit was assessed using the comparative fit index (CFI), standardized root mean square residual (SRMR), and root mean square error of approximation (RMSEA). Following the guidelines by Hu and Bentler^59^ and Browne and Cudeck^60^, CFI values greater than 0.95, SRMR values less than 0.08, and RMSEA values less than 0.05 were considered appropriate.

Eye-tracking data were preprocessed applying a velocity-based saccade detection algorithm^61,62^. Events with a velocity exceeding the average velocity during a trial by 6 median-based standard deviations for at least 6 data samples (i.e., 6 ms) were marked as saccades. Epochs between two subsequent saccades were defined as fixations. Single fixations and saccades were removed if they neighbored eye blinks or were outside the monitor area. If the first or last trial event was an ongoing saccade, it was also removed. For subsequent analyses, each fixation was assigned to one of two areas of interest: instructor (AOI-I) or material (AOI-M) (see Fig. 1b). We examined whether the allocation of attention between the instructor and additional sources of information was associated with the instructors’ level of immediacy using two three-way mixed-design analyses of variance (ANOVA). Outcome variables were the relative number of fixations on (H2a) and the relative total viewing time (H2b) directed at the instructor during part 1 (stimuli identical across all conditions) and part 3 (NVI levels differ across all conditions) of the videos. Part 2 was excluded from the analysis, as it only served to mask the changes in NVI levels throughout the videos. The relative number of fixations on the instructor was defined as the number of valid fixations within the target area (AOI-I) during each part of the video divided by the total number of valid fixations in the same time frame. Relative total viewing time directed at the instructor was defined as the quotient of the cumulated fixation durations within the target area divided by the total duration of fixations across the same time frame. In both models, experimental condition was included as a between-subject factor, while we controlled for video segments as a within-subject factor. Pairwise comparisons of group means were performed with a Games–Howell test^63^ adjusted for multiple testing using Tukey’s method. In addition, the relationship between the outcome variables and perceived NVI was examined using Pearson’s correlation coefficient. Statistical analysis was performed using R^64^.

### Ethics declaration

This study was performed in line with the principles of the Declaration of Helsinki and received approval from the University of Potsdam Ethics Committee (number 27/2023). Informed consent was obtained from all participants prior to participation. Individuals who are identifiable in the images and video gave informed consent for publication in an open-access online publication.

### Supplementary Information

Supplementary Information.

## Data Availability

The datasets generated during the current study are available from the corresponding author on^3^ reasonable request.
