# Supplementary material for: Exploring the impact of nonverbal social behavior on learning outcomes in instructional video design
Source: Sci Rep. 2024 Jun 4;14:12867. doi: 10.1038/s41598-024-63487-w (PMC11151426; doi:10.1038/s41598-024-63487-w)
Supplement: Supplementary file 1 — Supplementary Information. [file 41598_2024_63487_MOESM1_ESM.pdf]

Exploring the Impact of Nonverbal Social Behavior on Learning Outcomes in Instructional Video Design - Supplement

Jonas Frenkel[1,2,*], **Anke Cajar**[3], **Ralf Engbert**[3], **and Rebecca Lazarides**[1,2]

[1] Department of Educational Sciences, University of Potsdam, Karl-Liebknecht-Straße 24/25, 14476, Potsdam, Germany
[2] Science of Intelligence, Research Cluster of Excellence, Marchstraße 23, 10587 Berlin, https://www.scienceofintelligence.de
[3] Department of Psychology, University of Potsdam, Karl-Liebknecht-Straße 24/25, 14476, Potsdam, Germany
[*]Corresponding author: jonas.frenkel@uni-potsdam.de

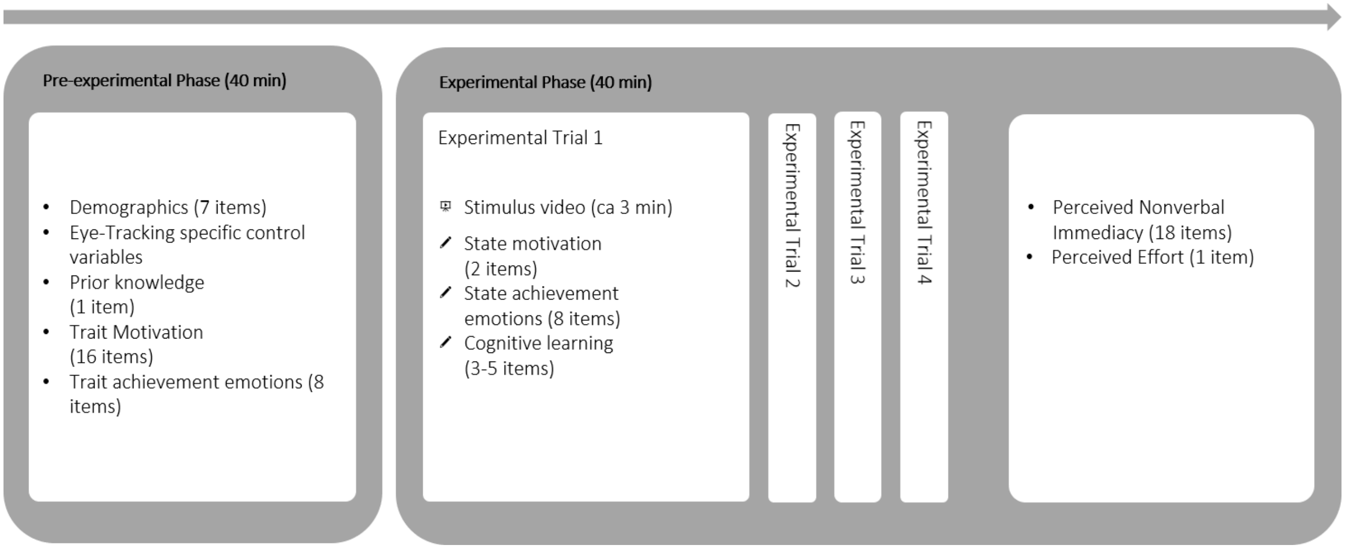

Figure S.1. Visual overview of the experimental procedure

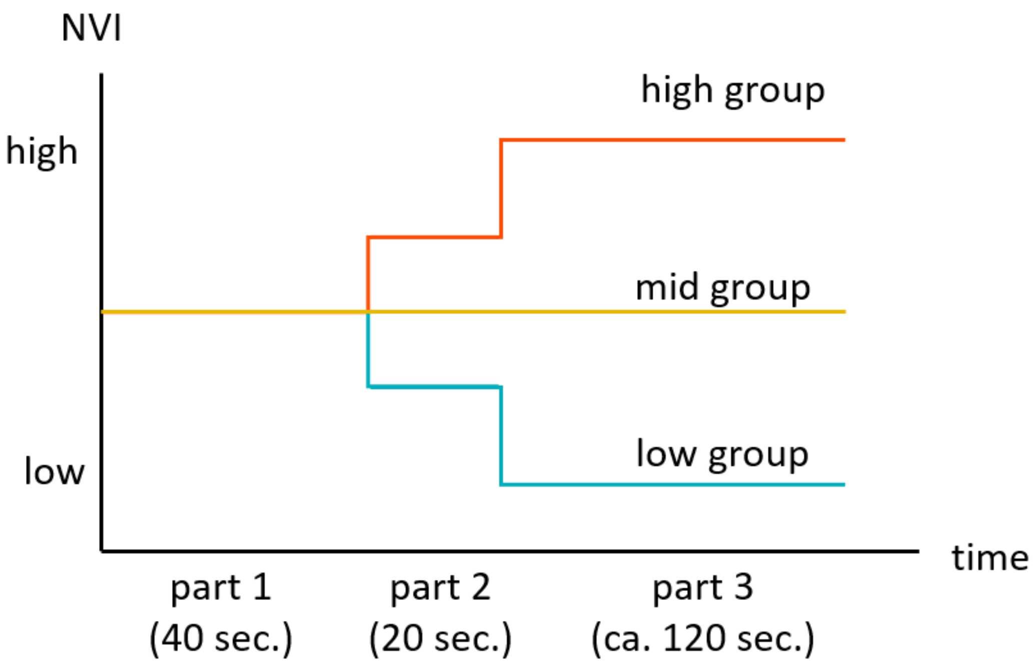

Figure S.2. Progression of displayed NVI in the experimental conditions over the course of each video. In the low and high conditions, a 40-second baseline phase with medium NVI is followed by a 20-second transition phase before the displayed NVI moves into the very high and very low ranges, respectively, for the remainder of the video. In the medium condition, the displayed NVI remains in the medium range for the entire course of the video.

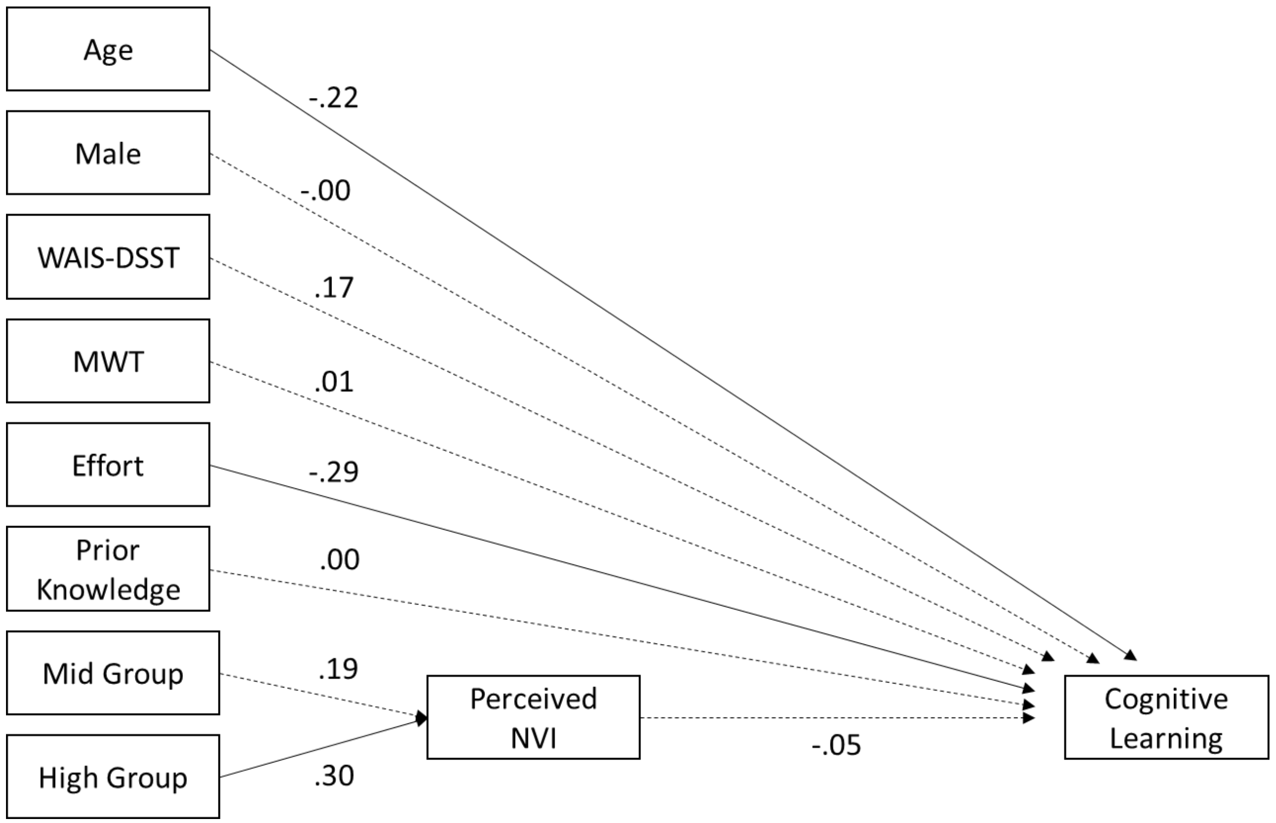

Figure S.3. Path model for the examined relationships associated with cognitive learning. Note. Solid lines indicate statistically significant ($p < .05$) standardized coefficients. Dashed lines indicate nonsignificant relations among constructs. NVI = Nonverbal Immediacy; WAIS-DSST = Wechsler Adult Intelligence Scale - Digit-Symbol subtest; MWT = Mehrfachwahl-Wortschatz-Intelligenztest (multiple-choice vocabulary intelligence test)

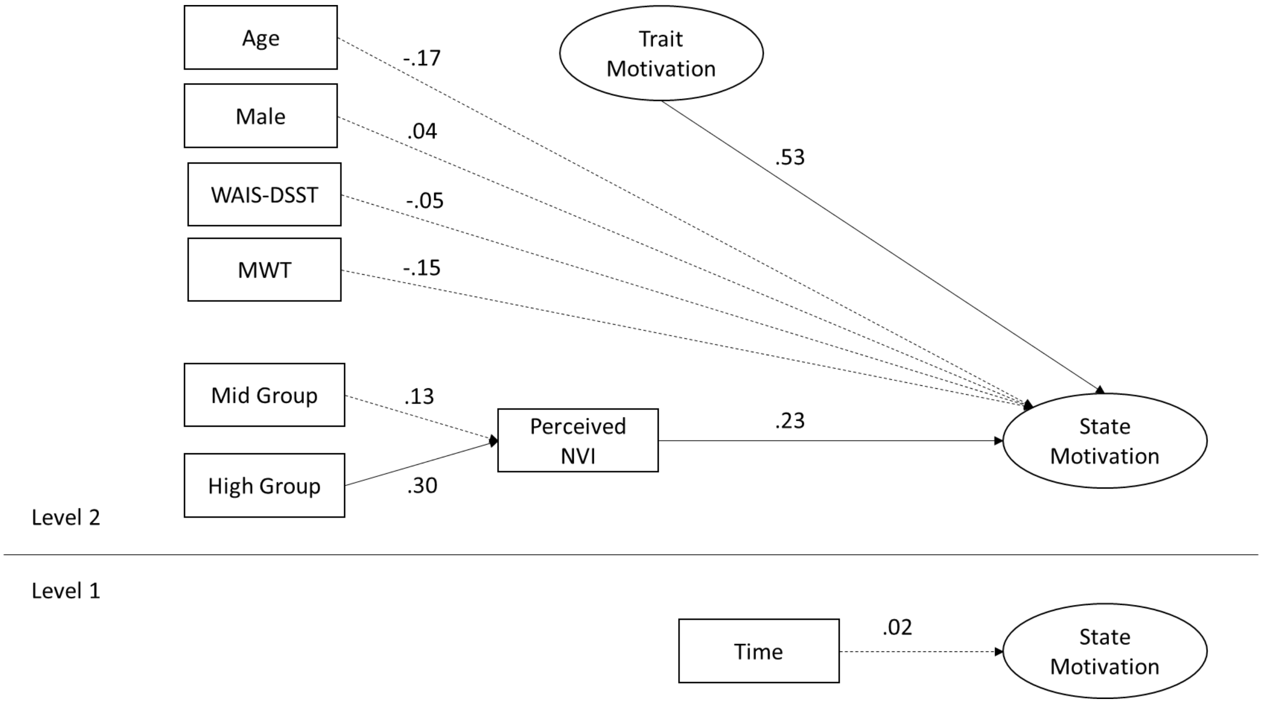

Figure S.4. Multilevel path model for the examined relationships associated with students' state motivation. Note. Solid lines indicate statistically significant ($p < .05$) standardized coefficients. Dashed lines indicate nonsignificant relations among constructs. NVI = Nonverbal Immediacy; WAIS-DSST = Wechsler Adult Intelligence Scale - Digit-Symbol subtest; MWT = Mehrfachwahl-Wortschatz-Intelligenztest (multiple-choice vocabulary intelligence test)

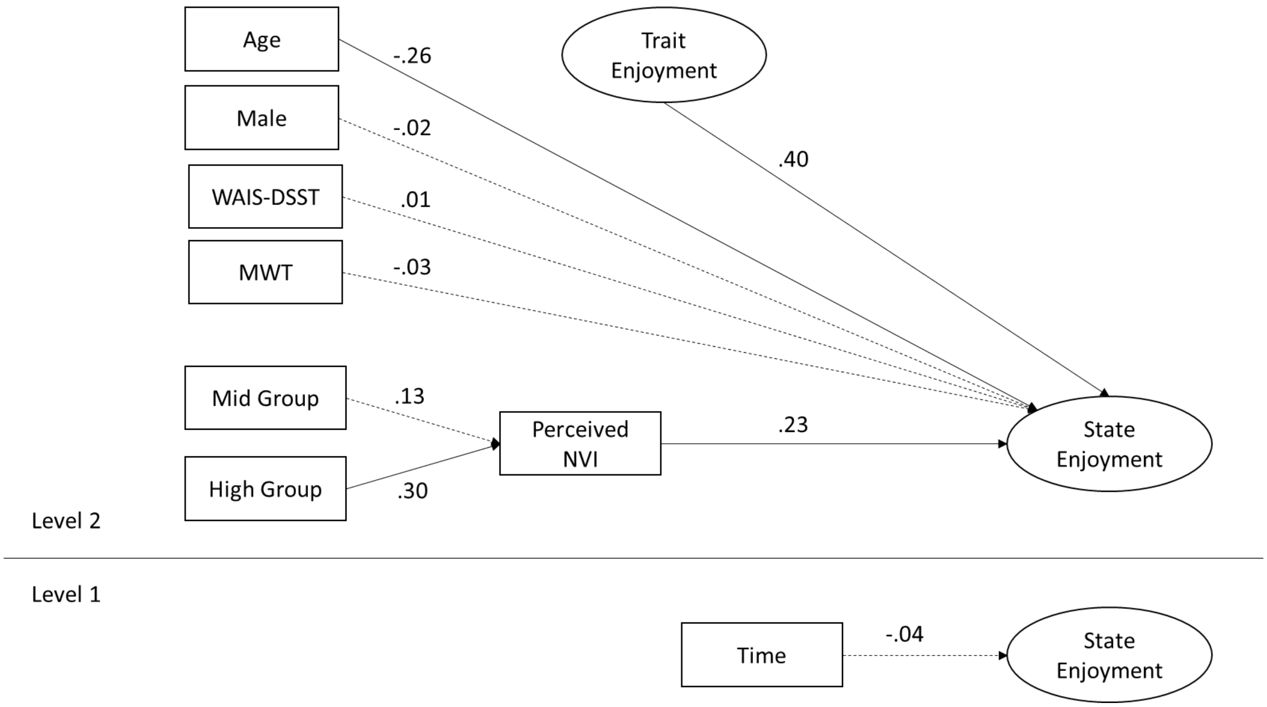

Figure S.5. Multilevel path model for the examined relationships associated with students' state enjoyment. Note. Solid lines indicate statistically significant ($p < .05$) standardized coefficients. Dashed lines indicate nonsignificant relations among constructs. NVI = Nonverbal Immediacy; WAIS-DSST = Wechsler Adult Intelligence Scale - Digit-Symbol subtest; MWT = Mehrfachwahl-Wortschatz-Intelligenztest (multiple-choice vocabulary intelligence test)

Video	Topics	Example Items
Video 1	• Introduction to the fundamental concepts and basic terminology<br>• Simple substitution cyphers: Caesar cipher	The science of encrypting and decrypting messages is also known as ___.<br>*Die Wissenschaft, die sich mit dem Ver- und Entschlüsseln von Nachrichten beschäftigt, nennt man auch ___.*<br>(A) Cryptography (B) Cryptonomy (C) Cryptography (D) Cryptanalysis<br>*(A) Kryptologie (B) Kryptonomie (C) Kryptographie (D) Kryptoanalyse*
Video 2	• Kerckhoff's principle<br>• Simple substitution cyphers: deranged alphabet and keyword usage	In (1), a fixed (2) is used instead of a shift number.<br>*Beim (1) wird anstelle einer Verschiebezahl eine feste (2) genutzt.*<br>(A) (1) Shifting method; (2) Secret table (B) (1) Replacement method; (2) Mapping table (C) (1) Kerkhoff method; (2) substitution table (D) (1) Assignment procedure; (2) Assignment number<br>*(A) (1) Verschiebeverfahren; (2) Geheimtabelle (B) (1) Ersetzungsverfahren; (2) Zuordnungstabelle (C) (1) Kerkhoffverfahren; (2) Ersetzungstabelle (D) (1) Zuordnungsverfahren; (2) Zuordnungszahl*
Video 3	• Differentiation between monoalphabetic vs. polyalphabetic cyphers<br>• Decryption by means of frequency analysis	The most common letters in the German language are:<br>*Die häufigsten Buchstaben in der deutschen Sprache sind:*<br>(A) E, O & A (B) E, T & O (C) U, K & N (D) E, N & I
Video 4	• Polyalphabetic cyphers: Vigenère cipher	In polyalphabetic cyphers, a letter of the plaintext is encrypted by ___ in the ciphertext.<br>*In polyalphabetischen Verfahren wird ein Buchstabe des Klartextes durch ___ im Geheimtext verschlüsselt.*<br>(A) several different letters (B) exactly one letter (C) Letters from 26 different alphabets (D) several randomly selected letters<br>*(A) mehrere verschiedene Buchstaben (B) genau einen Buchstaben (C) Buchstaben aus 26 verschiedenen Alphabeten (D) mehrere zufällig gewählte Buchstaben*

Table S.1. Overview of the topics presented in each of the videos and respective example items for assessing cognitive learning (original German items and English translation).

Nonverbal behavior	Low NVI condition	Medium NVI condition	High NVI condition
**Body posture**	Upright, stiff posture	Relaxed posture	Overtly relaxed and open posture, frequently shifting positions
**Facial expression**	low variety in facial expressions, predominantly neutral	Occasional smiling	Frequent smiling, high variety in expressions
**Gesture intensity**	Small, infrequent gestures, hands often clasped in front of the body	Frequent small gestures, isolated larger gestures, casual movements	Constant and expressive gestures, dynamic movements with arms open
**Tone of voice**	Monotone, slight variations	Moderate pitch and variation	Animated, expressive tone with varied pitch

Table S.2. Overview nonverbal behaviors displayed across the three experimental conditions.

	χ^2	df	CFI	ΔCFI	RMSEA	ΔRMSEA	$SRMR_{within}$	$\Delta SRMR_{within}$	$SRMR_{between}$	$\Delta SRMR_{between}$
State Motivation										
Configural invar	1.04	1	1.00		0.01		0.00		0.08	
Metric invar	0.54	2	1.00	0.00	0.00	-0.01	0.00	0.00	0.05	0.03
State Enjoyment										
Configural invar	1.82	4	1.00		0.00		0.02		0.00	
Metric invar	5.07	7	1.00	0.00	0.00	0.00	0.04	0.02	0.03	0.03

Table S.3. Model Fit Indices for Measurement Invariance Testing Across Levels and Time

Relative Number of Fixations on Teacher

	Total		Low condition		Mid condition		High condition	
	**M**	**SD**	**M**	**SD**	**M**	**SD**	**M**	**SD**
**All videos**								
Full videos	0.49	0.08	0.46	0.07	0.48	0.09	0.52	0.08
Individual parts								
Part one	0.52	0.10	0.51	0.10	0.50	0.11	0.56	0.10
Part two	0.42	0.10	0.39	0.10	0.42	0.10	0.44	0.11
Part three	0.52	0.08	0.48	0.06	0.52	0.07	0.57	0.08
**Video 1**								
Full video	0.57	0.10	0.53	0.08	0.57	0.12	0.61	0.08
Individual parts								
Part one	0.82	0.12	0.79	0.14	0.80	0.14	0.86	0.09
Part two	0.27	0.14	0.23	0.12	0.29	0.16	0.28	0.13
Part three	0.62	0.11	0.55	0.09	0.62	0.11	0.69	0.10
**Video 2**								
Full video	0.56	0.10	0.53	0.09	0.55	0.10	0.59	0.11
Individual parts								
Part one	0.38	0.12	0.36	0.11	0.35	0.11	0.41	0.12
Part two	0.74	0.17	0.73	0.17	0.74	0.16	0.75	0.20
Part three	0.56	0.09	0.51	0.08	0.56	0.08	0.60	0.10
**Video 3**								
Full video	0.44	0.10	0.42	0.09	0.43	0.09	0.46	0.11
Individual parts								
Part one	0.43	0.14	0.44	0.13	0.42	0.15	0.44	0.15
Part two	0.44	0.19	0.40	0.18	0.42	0.20	0.45	0.18
Part three	0.45	0.09	0.42	0.06	0.43	0.08	0.49	0.10
**Video 4**								
Full video	0.39	0.11	0.36	0.09	0.37	0.11	0.42	0.11
Individual parts								
Part one	0.47	0.16	0.45	0.13	0.44	0.17	0.52	0.17
Part two	0.23	0.14	0.22	0.13	0.24	0.15	0.24	0.14
Part three	0.46	0.09	0.42	0.06	0.45	0.08	0.51	0.09

Table S.4a. Descriptive statistics of the relative number of fixations on the teacher (AOI-A) for each experimental condition and video.

Relative Fixation Time on Teacher

	Total		Low condition		Mid condition		High condition	
	**M**	**SD**	**M**	**SD**	**M**	**SD**	**M**	**SD**
**All videos**								
Full videos	0.58	0.09	0.55	0.08	0.58	0.09	0.61	0.08
Individual parts								
Part one	0.65	0.10	0.63	0.21	0.64	0.22	0.67	0.21
Part two	0.50	0.11	0.47	0.26	0.51	0.27	0.51	0.26
Part three	0.59	0.08	0.54	0.11	0.58	0.12	0.64	0.12
**Video 1**								
Full video	0.65	0.09	0.61	0.09	0.66	0.11	0.68	0.08
Individual parts								
Part one	0.91	0.08	0.89	0.11	0.91	0.07	0.93	0.06
Part two	0.34	0.16	0.30	0.14	0.37	0.19	0.35	0.16
Part three	0.71	0.11	0.65	0.11	0.70	0.11	0.77	0.09
**Video 2**								
Full video	0.63	0.10	0.60	0.19	0.64	0.18	0.65	0.19
Individual parts								
Part one	0.49	0.14	0.46	0.14	0.48	0.13	0.52	0.14
Part two	0.80	0.16	0.79	0.14	0.81	0.14	0.79	0.20
Part three	0.61	0.09	0.56	0.09	0.61	0.08	0.64	0.09
**Video 3**								
Full video	0.54	0.11	0.52	0.11	0.53	0.10	0.56	0.12
Individual parts								
Part one	0.58	0.16	0.59	0.15	0.57	0.18	0.58	0.17
Part two	0.52	0.21	0.49	0.22	0.52	0.23	0.55	0.20
Part three	0.52	0.09	0.48	0.07	0.51	0.08	0.56	0.10
**Video 4**								
Full video	0.49	0.12	0.47	0.12	0.48	0.12	0.52	0.11
Individual parts								
Part one	0.62	0.16	0.59	0.17	0.60	0.17	0.67	0.14
Part two	0.32	0.19	0.31	0.18	0.33	0.22	0.32	0.18
Part three	0.53	0.09	0.49	0.09	0.52	0.09	0.57	0.09

Table S.4b. Descriptive statistics of the relative fixation durations on the teacher (AOI-A) for each experimental condition and video.
